# Geographical variation in case fatality rate and doubling time during the COVID-19 pandemic

**DOI:** 10.1017/S0950268820001685

**Published:** 2020-07-27

**Authors:** A. Mazumder, M. Arora, M. S. Sra, A. Gupta, P. Behera, M. Gupta, M. Agarwal, A. Rao, S. S. Mohanta, G. G. Parameswaran, A. Lohiya, H. D. Shewade

**Affiliations:** 1 All India Institute of Medical Sciences (AIIMS), New Delhi, India; 2 All India Institute of Medical Sciences (AIIMS), Bhubaneswar, India; 3 Indian Institute of Science Education and Research (IISER), Pune, India; 4 Super Specialty Cancer Institute and Hospital, Lucknow, Uttar Pradesh, India; 5 International Union Against Tuberculosis and Lung Disease (The Union), Paris, France; 6 The Union South-East Asia Office, New Delhi, India

**Keywords:** Coronavirus, epidemiology, public health

## Abstract

Case fatality rate (CFR) and doubling time are important characteristics of any epidemic. For coronavirus disease 2019 (COVID-19), wide variations in the CFR and doubling time have been noted among various countries. Early in the epidemic, CFR calculations involving all patients as denominator do not account for the hospitalised patients who are ill and will die in the future. Hence, we calculated cumulative CFR (cCFR) using only patients whose final clinical outcomes were known at a certain time point. We also estimated the daily average doubling time. Calculating CFR using this method leads to temporal stability in the fatality rates, the cCFR stabilises at different values for different countries. The possible reasons for this are an improved outcome rate by the end of the epidemic and a wider testing strategy. The United States, France, Turkey and China had high cCFR at the start due to low outcome rate. By 22 April, Germany, China and South Korea had a low cCFR. China and South Korea controlled the epidemic and achieved high doubling times. The doubling time in Russia did not cross 10 days during the study period.

In December 2019, coronavirus disease 2019 (COVID-19), a respiratory illness caused by a novel coronavirus − severe acute respiratory syndrome-coronavirus 2 (SARS-CoV-2) originated in Wuhan, China. On 11 March 2020, COVID-19 was declared as a pandemic by the World Health Organization. By 3 May 2020, around 240 000 people succumbed to this disease [1]. Asymptomatic cases have also been reported, further complicating its diagnosis and understanding its transmission epidemiology [2].

Case fatality rate (CFR) and doubling time are important parameters for judging the extent, rate and severity of an outbreak like COVID-19. CFR is an effective measure of the lethality of the disease and doubling time is used to track the rate at which the outbreak is progressing. The methods that have been traditionally used for the calculations of these parameters show considerable variations during the early stage of an outbreak. Alternate methods suggested by Galvani *et al*. for severe acute respiratory syndrome overcame this problem [3].

Traditionally, CFR is calculated by dividing the number of deaths due to a disease by the total number of individuals detected with the disease. While this method is reasonably accurate for an advanced epidemic, it yields inaccuracies in the early stages of the epidemic; particularly when the time of recovery or death is comparable to the duration of the epidemic. This method underestimates the CFR because it does not factor in the currently ill patients who will die in the future. Hence, we propose the calculation of cumulative CFR (cCFR) by excluding those who are still on treatment from the denominator. The cCFR in our analysis was calculated by dividing the total number of deaths at any time point by the sum of deaths and recoveries at that time point [3].
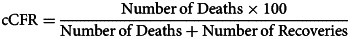


Another parameter, doubling time (the time required for the total number of cases in an epidemic to double) is a fundamental characteristic of an epidemic and is a function of the effective reproductive number (*R_t_*) and the serial interval (*v*). Doubling time indicates the rate of spread of the disease and the magnitude of control efforts required to curtail it. Moreover, it changes significantly throughout the epidemic. We have calculated the daily average doubling time by dividing the length of the time period in question by the log_2_ of the relative growth in the numbers of reported cases during the same period [3].
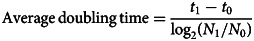
where *N*_1_ and *N*_0_ are the number of cases at times *t*_1_ and *t*_0_, respectively, where *t*_1_ and *t*_0_ represent consecutive days and hence the numerator is always 1. The units correspond to those used to measure the interval length *t*_1_−*t*_0_. We chose to plot the three-day moving average of the daily doubling time to smoothen the plot.

The outcome rate of a country is the fraction of cases for which the final outcome has been reported, be it death or recovery. It was calculated by dividing the number of patients who had a final outcome by the total number of known cases at any time point.
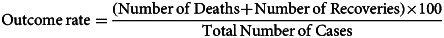


We assessed the fatality and spread of the disease by studying the variation in cCFR and doubling time over a specified period and compared them across various countries. Data were taken from 11 countries (China, South Korea, Italy, Spain, the United States, UK, Turkey, Russia, France, Germany, Iran and India) from publicly available data provided by Johns Hopkins University from 11 March 2020 to 22 April 2020 [4]. These countries were chosen because they were the top 10 countries (at the time of our analysis) based on the number of confirmed COVID-19 cases with sufficient progression into the outbreak (exhibiting a high outcome rate). South Korea was chosen as an interesting case study because of the apparent control of outbreak.

After plotting the values for cCFR and doubling time of all the countries (Fig. 1), we found that a country had either sufficiently progressed into the outbreak to show a relatively stable cCFR or was at the beginning of the outbreak and exhibited variation in the values. The United States, France, Turkey and China exhibited relatively high CFR in the beginning primarily due to their low outcome rate. At the beginning of an outbreak, deaths are more likely to be reported compared to recovery leading to an overestimation of the CFR; which decreases and stabilises as the outcome rate increases and more recoveries get reported. A higher outcome rate would result in the cCFR more closely estimating the CFR. This is validated by the fact that China shows an inverse relationship between outcome rate and cCFR (data not shown). These countries have a relatively high outcome rate towards the end of the observation period and show a uniform trend in the cCFR. The cCFR of all the countries shows a consistently lower value in the later phase compared to the starting possibly due to reporting of a greater number of recoveries. By 22 April, Germany, China and South Korea had a low cCFR. Another reason for the decrease in cCFR can be an improvisation in the testing strategy to include testing of asymptomatic individuals which are added ultimately to the recovery pool.

**Fig. 1.**
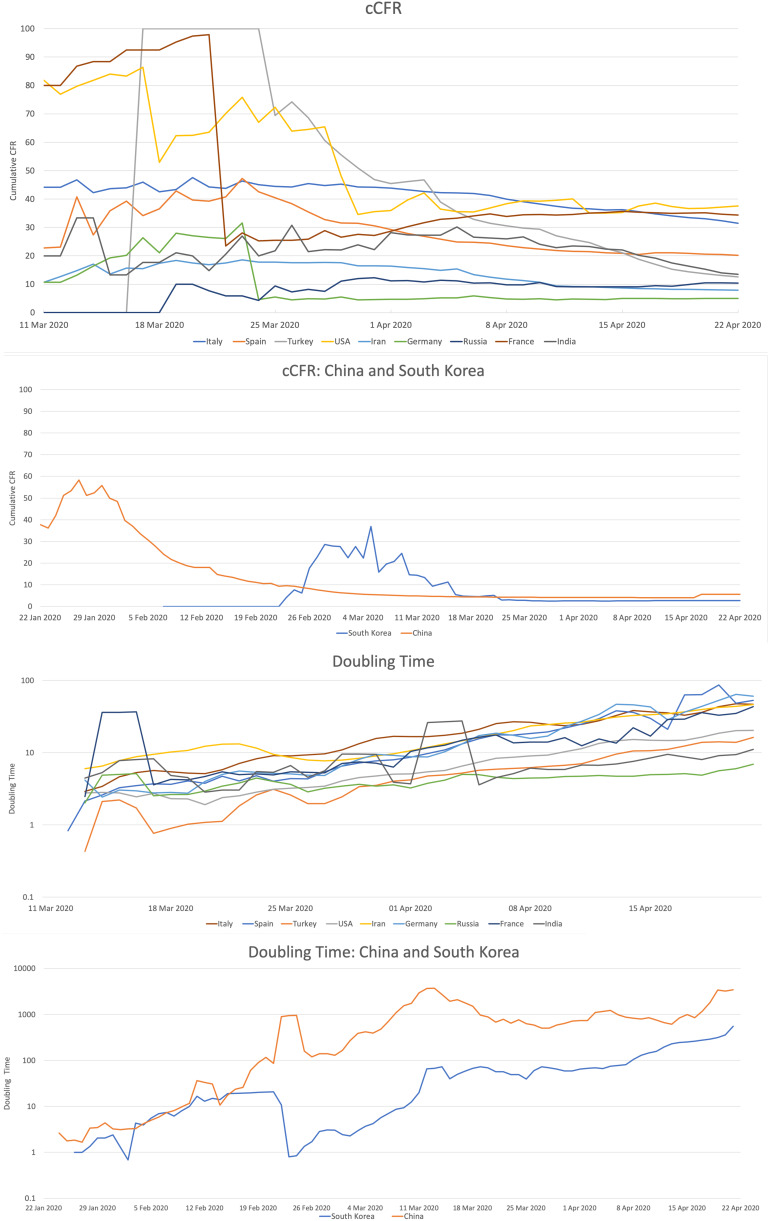
Trends of COVID-19 cCFR and doubling time for the countries included in our study (China and South Korea shown separately)*. cCFR, cumulative case fatality rate. *11 March to 22 April 2020 (all other countries), 22 January to 22 April 2020 (China and South Korea).

Since different ascertainment rates (the percentage of positive cases actuallybeing reported) in different countries may have an impact on cCFR, we plotted the cCFR for different countries (data notshown) against their cumulative test positivity rate (cTPR) as on 22 April 2020 (for all the countries except China whose datawere not available) [5]. An increased cTPR is indicative that testing is inadequate and more cases are missing detection (decreasing ascertainment rate). The analysis showed that there is a correlation between cCFR and cTPR with linear trend line (*R*^2^ = 0.304), where the cCFR for a country was more if its cTPR was more. The *R*^2^ increased to 0.624 if the data of Iran which was an outlier was removed (data not shown).

The cCFR and cTPR show a reasonable correlation, supporting the argument that countries with lower ascertainment rate have a higher cCFR. This may be because a lower ascertainment also usually means that clinically more severe cases are selectively detected to a higher extent, thus inflating cCFR estimates. However, detailed estimation of ascertainment rates need to be made to explicitly adjust the cCFR for the varying levels of underreporting across countries.

Over the same time, the doubling time has progressively increased in all the countries which indicates slowing down of the spread of COVID-19 due to better control efforts. China and South Korea controlled the epidemic and achieved high doubling times. On the other hand, the doubling time of Russia never crossed 10 days during the study period (Fig. 1). Transmission rate is indicative of the probability of disease transmission and is a function of the number of susceptible secondary contacts an infected individual has in his/her vicinity. This observation also has an important implication that during the early stages, for the prediction models which rely on calculation of basic reproduction number (*R*_0_), which by itself is based on the incubation period (*γ*), the duration of symptomatic period (*α*) and growth rate (*r*), the estimates should be used with caution [6–8]. This issue is compounded by asymptomatic carriers of COVID-19.

Since COVID-19 is a novel infection with no proven treatment or a protective vaccine, the mitigation and suppression strategies for containing the outbreak are primarily achieved through non-pharmaceutical interventions [9]. Suppression is aimed at breaking the chain of transmission and reducing the effective reproductive number (*R*_0_) to less than 1. On the contrary, mitigation is primarily aimed at delaying the spread of infection. These approaches are achieved through interventions like travel bans, lockdowns and stringent social distancing [10]. Even though majority of the countries have implemented similar measures, considerable heterogeneity in cCFR and doubling time may be due to the differences in the efficacy of contact tracing, the development of an accurate case definition and strategic testing.

Consistent with this explanation, China which has controlled the epidemic to a large extent using strict social distancing, quarantine and lockdown has a high doubling time and comparatively low cCFR. On the other hand, the United States which did not have lockdowns until much later has a low doubling time. The difference in the age structure of populations (higher mortality has been in older patients), the prevalence of various comorbidities, genomic mutation rates of the SARS-nCoV2, preparedness of the health system and prevalence of co-infection [11, 12] need to be considered for explaining the heterogeneity of CFR and doubling time worldwide. cCFR estimates may also decrease if the sensitivity of the test used increases which can occur if a country increases its PCR testing capacity. There would be more mild cases that would be detected and hence the cCFR estimates would fall as these mild cases are more likely to recover. There is also limited evidence to suggest that different strains may be present in different countries having variations in cCFR between strains [13]. There is a need to focus on broader issues such as standardisation of definitions, testing strategies and reporting format across countries to make more plausible comparisons. The global spread of COVID-19 mandates the need for international cooperation between nations, irrespective of the domestic status of the outbreak in order to prevent it from reappearing and causing a second wave of outbreaks across the world.

Our findings are limited by underreporting of infections, which may be due to lack of diagnostic tests. Underreporting is likely to be variable across countries as shown by the variable cumulative positivity rates. However, since accurate measures of the ascertainment rate could not be made, we could not account for variable ascertainment rate in our estimates. As long as the reporting remains invariant over time in a country, the calculation of doubling time remains constant, though this is a strong assumption. Alternatively, Russel *et al*. calculated a ratio of deaths-to-date by cases-to-date after adjusting for delays from confirmation of a case to death, and under-reporting of cases [14]. However, their estimates work on the assumption that the variations in the cCFR estimates among countries is only due to underreporting of cases which may not be true. By assuming that the actual CFR for all countries is 1.4%, their study fails to account for geographical variations shown by temporal stability of cCFR for different countries. The possible reasons discussed for these geographical variations should be studied further.

## Data Availability

Available data in the public domain were used for analysis and the source of data has been referenced.
